# Networks of High Mutual Information Define the Structural Proximity of Catalytic Sites: Implications for Catalytic Residue Identification

**DOI:** 10.1371/journal.pcbi.1000978

**Published:** 2010-11-04

**Authors:** Cristina Marino Buslje, Elin Teppa, Tomas Di Doménico, José María Delfino, Morten Nielsen

**Affiliations:** 1 Fundación Instituto Leloir, Buenos Aires, Argentina; 2 Institute of Biochemistry and Biophysics (IQUIFIB), School of Pharmacy and Biochemistry, University of Buenos Aires, Buenos Aires, Argentina; 3 Center for Biological Sequence Analysis, Department of Systems Biology, The Technical University of Denmark, Lyngby, Denmark; Columbia University, United States of America

## Abstract

Identification of catalytic residues (CR) is essential for the characterization of enzyme function. CR are, in general, conserved and located in the functional site of a protein in order to attain their function. However, many non-catalytic residues are highly conserved and not all CR are conserved throughout a given protein family making identification of CR a challenging task. Here, we put forward the hypothesis that CR carry a particular signature defined by networks of close proximity residues with high mutual information (MI), and that this signature can be applied to distinguish functional from other non-functional conserved residues. Using a data set of 434 Pfam families included in the catalytic site atlas (CSA) database, we tested this hypothesis and demonstrated that MI can complement amino acid conservation scores to detect CR. The Kullback-Leibler (KL) conservation measurement was shown to significantly outperform both the Shannon entropy and maximal frequency measurements. Residues in the proximity of catalytic sites were shown to be rich in shared MI. A structural proximity MI average score (termed pMI) was demonstrated to be a strong predictor for CR, thus confirming the proposed hypothesis. A structural proximity conservation average score (termed pC) was also calculated and demonstrated to carry distinct information from pMI. A catalytic likeliness score (Cls), combining the KL, pC and pMI measures, was shown to lead to significantly improved prediction accuracy. At a specificity of 0.90, the Cls method was found to have a sensitivity of 0.816. In summary, we demonstrate that networks of residues with high MI provide a distinct signature on CR and propose that such a signature should be present in other classes of functional residues where the requirement to maintain a particular function places limitations on the diversification of the structural environment along the course of evolution.

## Author Summary

Enzymes are responsible for several critical cellular functions. The so-called catalytic residues are fundamental to attain the enzyme function. Those residues are often highly conserved within protein families sharing similar structure and function. Characterization of catalytic residues is essential for the understanding of enzyme function. However, this is a difficult task because conservation is a poor discriminator of catalytic residues due to the fact that many non-catalytic residues are highly conserved in a given protein family. We anticipate that variations in the structural environment of a catalytic site should be highly restrained in order for the protein to maintain its function along the course of evolution, and hypothesise that catalytic residues, due to these restrains, must carry a particular signature defined by networks of proximity sharing high mutual information (MI). We validated this hypothesis on a large data set of protein sequences with known catalytic residues, and demonstrated that catalytic sites are indeed surrounded by networks of coevolved residues. Such networks should also be present in other classes of proteins and we suggest that MI networks could be a novel feature of general importance beneficial for the prediction of functional residues.

## Introduction

Catalytic residues play a fundamental role in enzymes and are generally expected to be conserved and located in the functional site of proteins. Even though characterization of catalytic residues (CR) is critical for the understanding of enzyme function, their identification remains a daunting task. To guide the identification of CR, several computational approaches have been developed based on different principles. To cite some examples: catalytic site features, amino acid physicochemical character [1], conserved functional groups density [2], sequence analysis (conservation, patterns, conserved blocks along the sequence, evolution, entropy, among others) [3], [4], [5], [6], [7], [8], sequence and structure properties [9], [10], [11], evolution and 3D structure information [12], [13], [14], [15], neural networks [16], 3D structure combined with ionization properties of a residue and its vicinity in the structure [17] and combinations of several of the above mentioned [18]. Conservation is the natural and intuitive way to predict functional residues in proteins. However, many non-catalytic residues are highly conserved and conversely, not all CR are fully conserved throughout a given protein family. On the other hand, residues involved in coevolving networks have been postulated to be functionally important [19], [20], [21] and several studies have provided evidence that they are important for specificity or allosteric regulation [22], [23], [24].

The structural environment of an active site must be highly conserved in order for the protein to maintain its function during the course of evolution. This places strict limitations on the amino acid diversity in the proximity of an active site, and it therefore seems plausible to hypothesise that catalytic residues would carry a particular signature defined by a network of close proximity of residues with high mutual information.

Although earlier published methods have suggested a linkage between functionally important sites and neighbouring coevolving residue [21], [25], [26] at present, to the best of our knowledge, no method explicitly show how the presence of such coevolving residues can provide quantitative information useful for catalytic sites identification beyond what is captured by conservation. Several methods have been proposed for identifying specificity defining positions (SDP) aiming at locating positions that are specific for a given subfamily and hence potentially could define its specificity [25], [26]. These residues are suggested to be located in the proximity of the active residues in order to carry out their role of defining the substrate specificity. The signal from such evolutional signatures could at first resemble co-evolution, and the overlap between the methods predicting SDPs and the method proposed here could seem substantial. However, the subfamily specific positions may not be coevolving, in fact they might be fully conserved within each subfamily, and Gouveia-Oliveira and Pedersen have described in details that such subfamily defining residues do not carry signatures of co-evolution but rather a phylogenetic signal that mimics coevolution [27]. The methods put forward by Gouveia-Oliveira et al, [27], Dunn et al. [28], and Buslje et al. [29] all attempt to reduce this phylogenetic bias in the signal for MI calculation aiming at identifying truly coevolving residue-pairs. Moreover, the method proposed here is hypothesis-free, and can be applied without any prior functional cluster classification of the input multiple alignment.

Here, we perform a large-scale benchmark analysis aiming at testing the hypothesis that catalytic residues carry a signature defined by networks of close proximity of residues with high mutual information. An investigation on the relationship between conservation, coevolution networks and catalytic residues is carried out on a dataset of 434 families of enzymes. We introduce a new concept, Mutual Information Proximity (pMI) that characterizes the mutual information network in the proximity of a given residue and analyse whether this measurement can complement the conventional conservation score for the detection of catalytic residues. The goal of this work is two-fold. First, we aim to validate the hypothesis stated above and demonstrate that proximity residue networks of high mutual information characterize functional residues. In doing this, we also aim at addressing the issue on the correlation between residues defined as SDP and residues carrying high signals of being part of the mutual information network. Secondly, we seek to integrate this mutual information signature to create a method able to identify catalytic residues useful for guiding the identification of functional sites in proteins.

Note, that in this work, we do not suggest that the proposed method should be more accurate than the other methods developed earlier for prediction of functional residues. We merely seek to demonstrate the existence of a mutual information network signature in the proximity of functional residues, and show that this signature is complementary to the conventional sequence conservation measurement, hence most likely would benefit any functional residue prediction method.

## Results

The main focus of this work was to investigate if mutual information could contribute beyond sequence conservation to the identification of catalytic residues. The result section naturally falls in three parts. First, we investigated how different measurements of sequence conservation could be used for the identification of catalytic residues. Next, a similar analysis was performed using different measurements of mutual information, and finally the analysis was carried out using a combined measurement of conservation and mutual information. Performance details of all methods included in the analysis are shown in supplementary table S2.

### Sequence conservation

As catalytic residues are highly conserved, a natural measure used to detect them is the conservation score in a MSA. Here, we investigated three conservation measurements in four different conditions leading to twelve different conservation scores (for details see material and methods). The conservation measurements are all per-residue measurements, and their predictive performance for a given protein sequence is readily measured in terms of the AUC value. The results of this analysis on the 434 CSA Pfam families are shown in table 1.

**Table 1:** Average performance in terms of the AUC and AUC01 values of the three methods: Max-Freq, Shannon, and Kullback-Leibler described to measure conservation.

Conservation measure	Max-Freq		Shannon		Kullback-Leibler	
	AUC	AUC01	AUC	AUC01	AUC	AUC01
Raw	0.874	0.458	0.880	0.464	**0.892**	0.485
C	0.870	0.461	0.876	0.465	0.890	**0.502**
L	0.857	0.380	0.852	0.371	0.877	0.437
Cl	0.847	0.353	0.837	0.335	0.868	0.411

Each measurement is applied under four conditions defined by sequence weighting using clustering (c); pseudo count correction using low counts (l), the combination of the two (cl), and no correction (raw). In bold is highlighted the method with the highest performance for each performance measure.

The conservation measurement with the highest predictive performance in terms of AUC was the raw KL score with an average AUC value of 0.892 and an AUC01 value of 0.485. In terms of AUC, the raw calculation excluding both sequence weighting and pseudo count correction did perform best for all three conservation measurements. In terms of AUC01, the inclusion of sequence weighting in all cases did improve the predictive performance. The Max-Freq measurement performed significantly worse than both information-based measurements (p<0.0001, binomial test excluding ties). Although the performance is very similar between the raw Shannon and raw KL scores, the difference is highly significant (p<0.005, binomial test excluding ties). The difference between the raw and sequence weighted (c) KL score is borderline significant with a p-value of 0.05 in favour of the raw KL score for AUC and in favour of KL including sequence weighting when using AUC01. In order to make the subsequent analyses as simple as possible, for the remaining part of the work we used the raw KL score as a conservation measurement.

**Figure 1:**
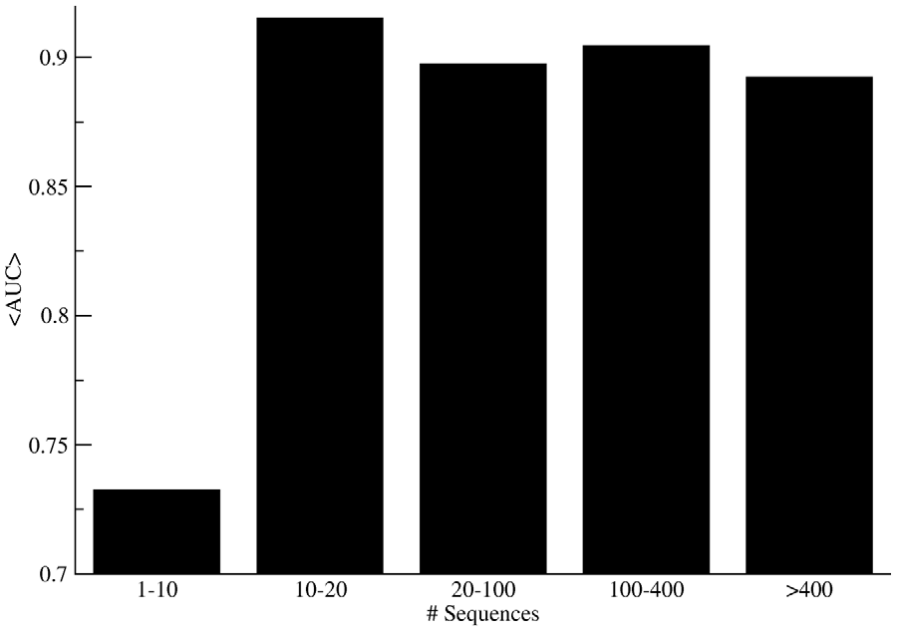
Histogram over predictive performance of the raw KL scores as a function of the number of sequences in the MSA. The number of Pfam entries in each sequence bin is 9, 9, 36, 66, and 314, respectively.

We analysed to what degree the predictive performance of the raw KL measurement depended on the number of sequences in the multiple sequence alignment (MSA) used as the source to estimate the conservation score (see figure 1). This figure clearly demonstrates that at least 10 sequences are required in order to make any meaningful predictions using the KL conservation measurement (similar results were observed for the other two conservation measurements). Note, that the variation in performance for each bar in the histogram is large and error-bars are not included (the raw data included in the figure are available in Supplementary table S2). The difference in predictive performance between the families with less than or more than 10 sequence members is however statistically highly significant (p<0.001, t-test).

### Mutual information

**Figure 2:**
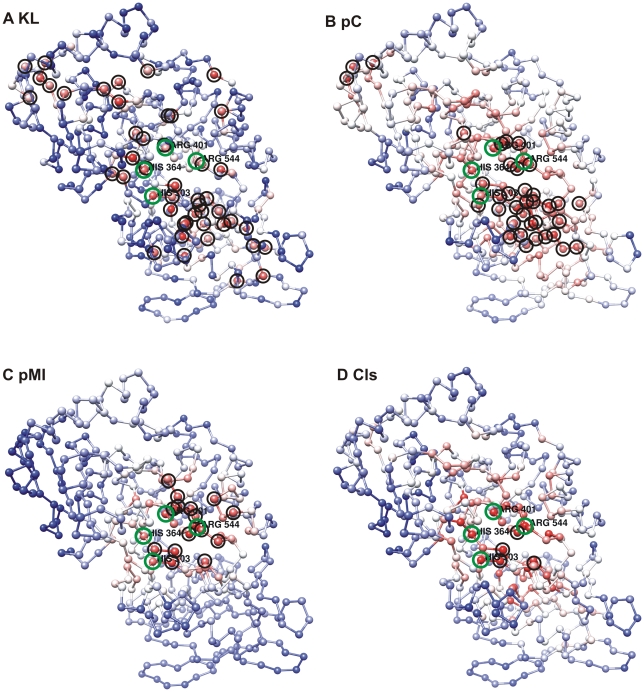
Identification of catalytic residues using four different prediction scores. Plotted is the Cα representation of the PDB entry 1D4C representing the Pfam PF00890 entry. Catalytic residues are encircled in green. The four different prediction scores are shown A) KL Conservation, B) Proximity conservation (pC), C) proximity MI (pMI) and D) Catalytic likeliness score (Cls). Highlighted with black circles are the predicted false positive residues: 47, 39, 15 and 4 respectively. The prediction scores are represented in blue to red scale (blue: lowest; red: highest). Molecular graphics image was produce with UCSF Chimera package. (University of California, San Francisco).

We next turned to mutual information and analysed the environment of a catalytic residue by means of the mutual information carried by the surrounding residues. We introduced a cumulative Mutual Information concept (cMI) that measures the degree of shared mutual information of a given residue (above a certain significance threshold as measured in terms of the MI Z-score, see material and methods). We noticed that residues in close proximity with CR tend to have high cMI scores (see figure 2b). Furthermore, when measuring the proximity Mutual Information (pMI), which tells about the networks of mutual information in the proximity of a residue (within a certain distance threshold), the catalytic residues were observed to have higher pMI than other conserved residues (see figure 2c for an example).

We exploited this observation on the complete Pfam benchmark dataset, and calculated the performance of the pMI measurement as a predictor of catalytic residues. Using a distance cut-off of 7.5 Å to define the structural proximity, and a Z-score threshold of 6.0 to define reliable mutual information interactions (see [29]), the average predictive performance of the pMI measurement in terms of the average AUC and AUC01 values on the 434 Pfam entries was 0.843 and 0.342, respectively which in both cases is significantly different from random (p<0.0001, binomial test excluding ties). As the number of proximity interactions is used to normalize the pMI measurement, this predictive performance does not stem from any implicit bias in the data imposed by catalytic residues being in a particular state of solvent exposure.

### Comparison between SDPs and cMI

To investigate how the mutual information measure (cMI) proposed in this work correlates to earlier proposed measures for SDP, we compared in terms of the Spearmans rank correlation the SDR Z-score values given in the SDR database (http://paradox.harvard.edu/sdr/) [30] to the cMI values. In doing this, we obtained a mean correlation value over the 158 Pfam families covered by both methods of 0.29+/−0.20 (for details see materials and methods). Even though this correlation is significantly different from random (p<0.01, binomial test excluding ties), it is far from perfect. This highly suggests that the cMI and SDR measures carry distinct information. We next calculated the correlation between the two measures and the KL (Kullback-Leibler) conservation score. Here, we obtained an average Spearmans rank correlation values of 0.64±0.21, and −0.04±0.17 for the SDR Z-score and cMI measure respectively. These results further demonstrate that the SDP and cMI measures are different in nature, and that SDR Z-score is highly related to sequence conservation whereas the cMI score is independent of the latter. This strongly suggests that the cMI measure is more information rich compared to SDP when combined with sequence conservation.

### Conservation of the residue proximity

As the active site in most cases is defined in terms of multiple catalytic residues in close proximity, it is natural to suggest that a proximity score based on sequence conservation would be a strong catalytic residue predictor. Using the same distance cut-off as for the mutual information proximity score, we find that the proximity conservation score, pC, achieves an average predictive performance of 0.854 and 0.379 in terms of AUC and AUC01, respectively. These values are greater than what was obtained using the pMI score, but for both AUC and AUC01, the difference between the two methods is not statistically significant (p<0.05, binomial test excluding ties).

### Combined catalytic likeliness score

We finally applied the combined catalytic likeliness score (Cls) to identify catalytic residues. The Cls is calculated as a weighted sum of the KL conservation the pMI mutual information and the pC scores. The optimal parameters defining the score were identified using 5-fold cross validation as described in Materials and Methods. The parameters Z_thr_, D_MI_, D_C_, W_MI_ and W_C_ were found to have the following optimal values Z_thr_ = 5.5±0.2, D_MI_ = 8.0±0.1, D_C_ = 5.6±0.5, w_MI_ = 0.6±0.0, and w_C_ = 0.2±0.0. The low standard deviation value on each parameter-estimate indicates that the parameter optimization is robust across the different cross-validation data sets. The average performance in terms of the AUC and AUC01 of the Cls score to detect catalytic residues was 0.927, and 0.594, respectively. This performance is significantly higher than the KL conservation, the pMI and the pC individual scoring functions (p<0.001 in all cases using binomial test excluding ties).

To investigate the individual contribution to the performance of the Cls score of the pMI and pC measures, we next searched for optimal parameters for a combined score including only one of the two proximity measures in combination with the KL conservation score. Estimating the optimal parameters using 5 fold cross-validation as described above, we find the following results (see table 2).

**Table 2:** Optimal parameters and average predictive performance in terms of AUC and AUC01 for the two combined prediction methods including only one proximity measure.

Method	KL+pMI	KL+pC
Parameters	w_MI_ = 0.8±0.0	w_C_ = 0.6±0.0
	D_MI_ = 7.9±0.2	D_C_ = 8.0±0.0
	Z_thr_ = 5.5±0.32	
AUC	0.922	0.910
AUC01	0.574	0.562

KL+pMI is the method combining KL conservation with the pMI mutual information measure. KL+pC is the method combining KL conservation with the pC conservation measure. w_MI_ is the relative weight on pMI, D_MI_ is the proximity distance threshold for the pMI measure, Z_thr_ is the MI Z-score threshold, w_C_ is the relative weight on pC, and D_C_ is the proximity distance threshold for the pC measure. Parameters and standard deviations were identified using five-fold cross validation as described in Materials and Methods.

The AUC values for both of these methods are significantly lower that what was obtained using the Cls score combining the conservation score with both proximity measures (p<0.01 in both cases, binomial test excluding ties) demonstrating that the two proximity measures contribute distinct information to the combined Cls score. The difference between the two scores including only one proximity measure is not statistically significant when looking at the complete data set of 434 PF families. However, when looking at the subset of 172 PFam families that are covered by more than 400 unique sequences/clusters (corresponding to the number of clusters needed to provide reliable estimates of MI as shown by Buslje et al. [29]), the combined method including proximity mutual information, pMI, achieves a performance of AUC = 0.920, and AUC01 = 0.597. These values significantly outperform the performance values AUC = 0.889 and AUC01 = 0.559 of the combined method including proximity conservation, pC (p<0.05, binomial test excluding ties). This further underlines the observation that the pMI measure contributes information not included in the conservation scores.

To further illustrate that the two proximity measures contribute different information to the combined Cls-score, we in figure 2 display the role of the four prediction measurements, KL, pMI, pC and Cls for the identification of the catalytic residues in the Pfam entry PF00890 represented by fumarate reductase of Shewanella putrefaciens MR-1 (PDB entry 1D4C). This family was chosen from the subset of 172 Pfams entries mentioned above covered by more than 400 unique sequences/clusters (similar results are obtained for most other families in this set). The function of fumarate reductase is carried out by the active cite residues His364, Arg401, His503 and Arg544 [31]. It can be seen that the KL conservation score of the catalytic residues is relatively low (figure 2a) while both the pC, and pMI scores are high in the catalytic residue proximity (figure 2b, and 2c). Comparing the figures 2b and 2c, it is evident that the two proximity measures contribute different information to the combined, Cls, prediction score. Finally, the combined catalytic likeliness score (Cls) is depicted in figure 2d. The AUC values for the four prediction measurements shown in figure 2 are 0.92, 0.94, 0.98 and 0.99 (KL, pC, pMI and Cls respectively). These values translate into a number of false positive predictions at 100% sensitivity (corresponding to the number of non-catalytic residues with a prediction score higher than the lowest score obtained by a CR) of 47 (figure 2a), 39 (figure 2b), 15 (figure 2c), and 4 (figure 2d), again underlining the strong predictive power of the Cls measurements in identifying catalytic residues and eliminating false positive predictions.

The gain in predictive performance for detecting catalytic residues is consistent for families independently on the level of conservation of the catalytic residue, however the most dramatic gain in performance when including pMI is observed for families where the conservation of the catalytic residues is poor. If we for instance take the 217 Pfam families with the lowest predictive performance when using the KL conservation score and ask how many of these families gain in performance when including the pMI score, we find that this number is significantly higher compared to the corresponding number of families in the group of 217 Pfam families with the highest predictive performance using the KL conservation score (p<0.001, binomial test excluding ties). This difference in performance gain between the two subsets of Pfam families is not imposed by a difference in data size between the two sets as the average family size in the two set is comparable (p>0.1, t-test). The catalytic environment of an active site needs to be conserved in order for a protein family to maintain its function, and one might speculate that when the conservation of a catalytic residue is weak, the catalytic environment is maintained in great measure by coevolution.

We next determined the sensitivities of the different methods at different specificity thresholds. This analysis is summarized in table 3. The analysis clearly confirms the strong improvement across the entire benchmark data set of the predictability of catalytic residues imposed by the inclusion of the pMI score in the combined catalytic likeliness score. At all specificity thresholds, the Cls method did achieve the highest sensitivity. The difference in sensitivity between the Cls and the other methods is statistically significant (p<0.05, binomial test excluding ties) for all comparisons. The Cls score threshold corresponding to a specificity of 0.90 for the 434 CSA families is 1.44±0.26. This low standard deviation of the threshold score indicates that the Cls approach is stable across the different CSA families and suggests that the method can be applied universally to any enzyme protein family independently of diversities in structure, composition and size of the MSA, as long as the number of sequences is greater than 10 (see figure 1).

**Table 3:** Sensitivity of the catalytic residue identification methods at different specificity thresholds.

	Sensitivity					
Specificity	KL	pMI	pC	KL+pMI	KL+pC	Cls
0.99	0.222	0.122	0.159	0.300	0.282	**0.315**
0.95	0.544	0.375	0.423	0.646	0.637	**0.667**
0.90	0.716	0.560	0.604	0.802	0.774	**0.816**
0.85	0.798	0.666	0.703	0.861	0.835	**0.862**

KL is the Kullback-Leibler conservation score, pMI is the proximity averaged mutual information score. pC is the proximity averaged conservation score, KL+pMI is the combined score of KL and pMI, KL+pC is the combined score of KL and pC, and Cls is the Catalytic likeliness score, The sensitivity is determined as an average over the 434 CSA families at the different specificity thresholds. In bold is highlighted the best performing method at each specificity level.

## Discussion

Catalytic residues are in general expected to be conserved and located in the functional site of a protein in order to attain their function. However, many non-catalytic residues are highly conserved as well and conversely, not all catalytic residues are conserved throughout a given protein family, making identification of catalytic residues a big challenge. The requirement to maintain a given catalytic function during the course of evolution places great limitations on the diversity of the structural environment of an active site. Therefore, here we put forward the hypothesis that catalytic residues carry a particular signature defined by networks of close spatial proximity residues sharing high mutual information, so that this signature could be applied to differentiate functional from other non-functional conserved residues.

We tested this hypothesis using a data set of 434 Pfam families each characterized by a PDB structure and one or more catalytic residues assigned from the CSA database, and investigated whether mutual information could complement conventional amino acid conservation scores and improve the ability to detect catalytic residues. Three methods to calculate sequence conservation were considered and the KL relative entropy (KL) was shown to significantly outperform both the Shannon entropy and maximal frequency measurements. We observed that sequence-weighting and low count correction do not improve the predictive performance for any of the methods. Additionally, in order to achieve reliable predictions the number of sequences required in the MSA was found to be relatively small. Only 10 sequences in the MSA were needed to reach AUC values of 0.89.

We observed that in the proximity of a catalytic site, residues are rich in shared mutual information (calculated as the cumulative mutual information, cMI): therefore, we defined a residue specific score characterizing this fact in terms of a structural proximity average (termed pMI) score. The pMI score was demonstrated to be a strong predictor for catalytic residues, suggesting that catalytic residues indeed carry a particular signature imposed by networks of mutual information. We compared the predictive performance of the pMI measure to that of a proximity measure based on sequence conservation and demonstrated that the two measures achieved comparable predictive performance but more importantly that they carried distinct information suitable as predictor of catalytic residues. Finally, we demonstrated that the conventional KL relative entropy sequence conservation, the pC and pMI measurements are complementary and that a combined catalytic likeliness score (Cls) of the three leads to significantly improved prediction accuracy. For instance, we found that, at a specificity threshold of 0.90, the KL, pMI, pC and Cls methods have a sensitivity of 0.716, 0.560, 0.604 and 0.816, respectively.

This work thus demonstrates in direct quantitative terms (gain in predictive performance) the contribution of the coevolution signal in determining catalytic residues, and hence goes beyond earlier published papers in the field [20], [21], [25], [26] and not only describe the observation that such signals might be present near functionally important residues but in details demonstrate how such information can be applied to guide their identification.

We also analyzed to what extent the score characterizing specificity defining positions (SDPs) and the mutual information derived score defined in this work carry distinct information on the functional neighbor of catalytic residues. We used data from the Paradox database to carry out the comparison, and compared SDP and cMI scores for a set of 158 families covered by both methods. The obtained results clearly demonstrated that the SDP and cMI measures are different in nature, and that SDR Z-score is highly related to sequence conservation whereas the cMI score is independent of the latter. This observation strongly suggests that the cMI measure is more information rich for the identification of functional residues compared to SDP when combined with sequence conservation.

In summary, we have demonstrated that mutual information provides a distinct proximity signature that can be applied to determine catalytic residues. The approach outlined is general, and we suggest that the method should be applicable to the identification of other classes of functional residues where the requirement to maintain a particular function places limitations on the diversity of the structural environment along the course of evolution.

## Materials and Methods

### Dataset

The dataset was constructed based on the CSA database (version 2.2.11, released August 2009) [32]. CSA provides catalytic site annotation for enzymes in the PDB. Catalytic residues were defined as those residues thought to be directly involved in some aspect of the reaction catalysed by an enzyme (for a detailed description of the classification see [1]). The database consists of two types of annotated sites: an original, hand annotated set and an additional homologous set, containing annotations inferred by Psi-Blast and sequence alignment to one of the original entries. CSA contains 968 original literature entries, which belong to 455 Pfam families [33]. Due to some inconsistency between CSA and PDB, a few families were eliminated, so that we ended up with a dataset of 434 protein families (each of one containing at least one PDB entry), which in turn include a total of 1212 CSA, annotated catalytic residues. For 9 of the 434 families the selected PDB representative was an NMR structure. For these PDB entries the first model was selected to represent the structure. The 434 Pfam families included in the benchmark data set cover 8 SCOP classes, 199 folds, 249 super families and 389 families.

When more than one PDB entry with catalytic site annotation was available for a given family, one reference PDB entry was selected following the criteria: highest sequence coverage of the Pfam MSA, the year of structure determination (preferably later than 2000) and resolution (Supplementary table S1 provides the Pfam family and reference PDB). In all cases, MSAs were gap trimmed to remove positions with gaps in the reference sequence. In addition, all positions with >50% gaps, as well as sequences covering <50% of the reference sequence length were removed, as described in [29]. Supplementary figure S1 shows the distribution of the number of sequences and sequence clusters in the dataset.

### Conservation

Conservation of each position in the MSA's was calculated with three different measurements: Shannon entropy [34], KL relative entropy [35] calculated using an amino acids background frequency distribution obtained from the Uniprot database [36] and the maximal frequency (the frequency of the most represented amino acid). Each of these measurements were calculated from the raw MSA, from the MSA corrected for sequence redundancy using sequence weighting by 62% identity clustering (c), from the MSA including pseudo-counts to correct for low counts (l) [37], [38] and from the MSA applying both clustering and pseudo-count correction (cl). The total number of conservation measurements investigated was hence twelve.

### Mutual information

Mutual information (MI) was calculated as described in [29]. In short, the MI is calculated between pairs of columns in the MSA. The frequency for each amino acid pair is calculated using techniques of sequences weighting and low count corrections and is compared to the expected pair-frequency assuming that the amino acids are non-correlated. Next, the MI is calculated as a weighted sum of the log-ratios between the observed and expected amino acids pair frequencies. The APC method of Dunn et al. [28] was applied to reduce the background mutual information signal for each pair of positions and the MI scores were finally translated into MI Z-scores by comparing the MI values for each pair of position to a large set of MI values calculated from permutated MSA. MI gives a value for each pair of residues in a MSA. We sought a mutual information score per residue that characterizes the extent of mutual information “interactions” in its physical neighbourhood. This score was defined in two steps. First, we calculated a cumulative mutual information score (cMI) for each residue as the sum of MI values above a certain threshold for every amino acid pair where the particular residue appears. This value defines to what degree a given amino acid takes part in a mutual information network. Next, we defined a proximity average for each residue as the average of cMI of all the residues within a certain physical distance to the given amino acid. Finally, we normalized the proximity average values for a given MSA to fall in the range [0–1] to obtain the proximity MI (pMI) score. The distance between each pair of residues in the structure was calculated as the shortest distance between any two atoms different from H belonging to each of the two residues.

### Combined catalytic likeliness score

We define a combined catalytic likeliness score (Cls) as a weighted sum of the conservation (defined in terms of the KL relative entropy), the proximity mutual information (pMI) and the proximity conservation (pC) scores.
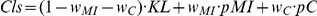
Here, pC is the average conservation score of residues within a given proximity distance, and w_C_, and w_MI_ are adjustable relative weights.

### Parameter optimization

The calculation of the combined catalytic likeliness score depends on three parameters; Z_thr_ (Z-score threshold for including an amino acids pair in the cMI score), D_MI_ (distance threshold to include an amino acid in the pMI average score), D_C_ (distance threshold to include an amino acid in the pC average score), and the relative weights, *w_MI_* and *w_C_*, on pMI and pC, respectively. These parameters were estimated using five-fold cross validation, where optimal values were obtained using brute force grid-sampling on 4/5 of the data set to optimize the average AUC value and the remaining 1/5 of the data was evaluated next using this set of optimal parameters. This procedure was repeated five times leading to five sets of optimal parameters and evaluation performance values for each MSA in the data set.

### Measurement of predictive performance

The predictive performance in detecting catalytic residues, by way of conservation, pMI and Cls, was evaluated in terms of the area under the ROC curve (AUC) [39] per family. The AUC measure might not be optimal if the benchmark data set has a high ratio on negative data, and a high specificity in actual number could translate into a large number of false positive. In such situations, it might be beneficial to use only the high specificity part of the ROC curve to calculate the predictive performance. Here, we hence complement the AUC measure with AUC01 calculated including only the specificity range for 1 to 0.9 when calculating the AUC. For both measures will a value of 1 indicate a perfect prediction while a value of 0.5 indicates a random prediction. Annotated catalytic residues in the CSA were taken as the positive set, and all other residues with annotated PDB-ATOM coordinates were assigned as negative. The final performance was determined as the average AUC over the 434 CSA Pfam families.

### Comparison between SDPs and cMI scores

We downloaded the entire Paradox SDR database (specificity-determining residues in protein families database; http://paradox.harvard.edu/sdr/), and identified the subset of families present in our benchmark dataset where the reference sequence from the CSA database was also member of the paradox multiple sequence alignment (MSA). This gave us a set of 158 families. The Paradox database provides SDR Z-scores only for a subset of the positions in the MSA [30]. Residues with undefined SDR Z-score were assigned a Z-score of 0 to allow for complete sequence coverage. Next, we compare for each family the SDR Z-score value to our cMI (cumulative mutual information) value of each position in the alignment in terms of the Spearmans rank correlation. We also calculate the Spearmans rank correlation between KL and both SDR Z-score and cMI values of each position for each family in the dataset.

## Supporting Information

Figure S1 Histogram of the number of families in the Pfam benchmark data set. A) number of sequences B)number of clusters. The insets show a zoom from 0 to 1,000 sequences/clusters.
(0.02 MB PDF)

Table S1 Pfam PDB correlation. Pfam accession, PDB taken as reference for that family, and pdb region included in the analysis.
(0.03 MB PDF)

Table S2 Performance details of all methods included in the analysis. Cons and C means conservation; pMI: proximity MI; pC: proximity conservation, Cls: catalytic likeliness score; Nseq: number of sequences; Ncluster:number of clusters; pdb: pdb taken as reference.
(0.16 MB XLS)
