# Supplementary figures and images for: Networks of High Mutual Information Define the Structural Proximity of Catalytic Sites: Implications for Catalytic Residue Identification
Source: PLoS Comput Biol. 2010 Nov 4;6(11):e1000978. doi: 10.1371/journal.pcbi.1000978 (PMC2973806; doi:10.1371/journal.pcbi.1000978)

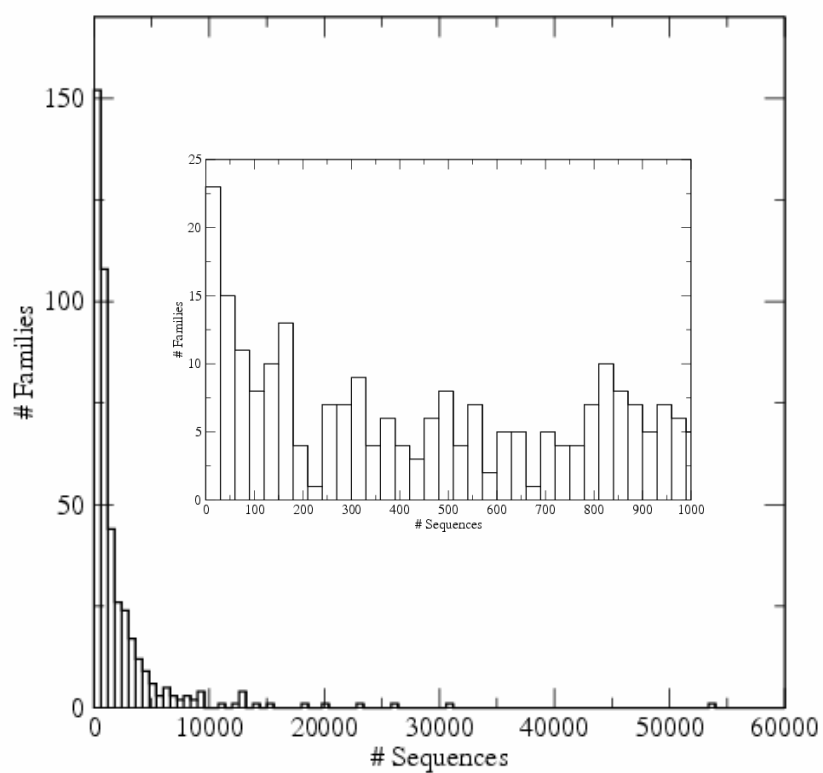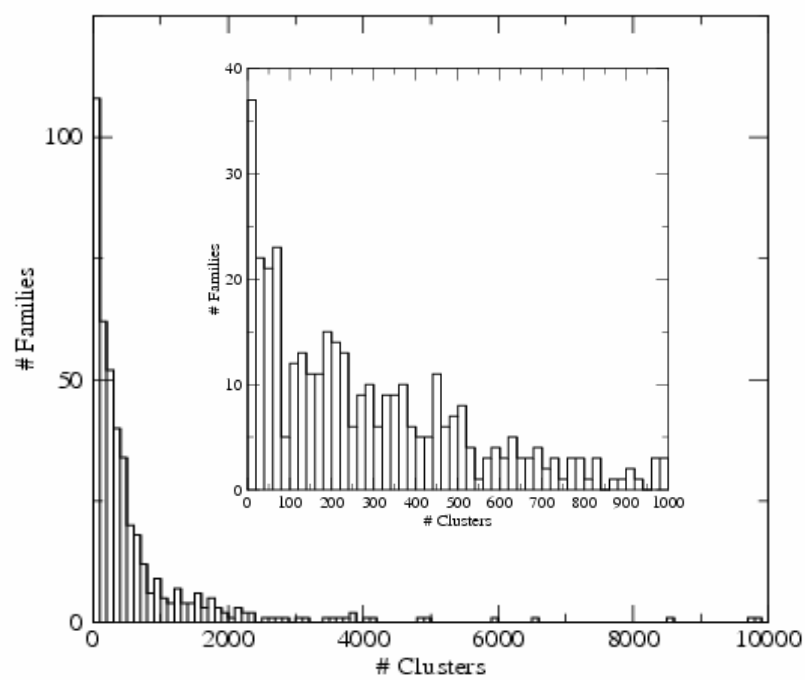

Supplement: Figure S1 — Histogram of the number of families in the Pfam benchmark data set. A) number of sequences B)number of clusters. The insets show a zoom from 0 to 1,000 sequences/clusters. (0.02 MB PDF) [file pcbi.1000978.s001.pdf]
