# Supplementary material for: Networks of High Mutual Information Define the Structural Proximity of Catalytic Sites: Implications for Catalytic Residue Identification
Source: PLoS Comput Biol. 2010 Nov 4;6(11):e1000978. doi: 10.1371/journal.pcbi.1000978 (PMC2973806; doi:10.1371/journal.pcbi.1000978)
Supplement: Table S1 — Pfam PDB correlation. Pfam accession, PDB taken as reference for that family, and pdb region included in the analysis. (0.03 MB PDF) [file pcbi.1000978.s002.pdf]

PFAM Accession	Uniprot Accession/residue numbers PDB code chain/ residue numbers
PF00085	PDIA1_HUMAN/25-132 PDB: 1mek / 8-115
PF00004	NSF_CRIGR/538-571 PDB: 1nsf / 538-571
PF00006	VATA_YEAST/236-916 PDB: 1lws A/ 1-454
PF00009	EFTU_ECOLI/10-202 PDB: 1d8t A/ 10-202
PF00012	HSP7C_BOVIN/6-612 PDB: 1kaz / 6-381
PF00016	RBL_SYNP6/151-459 PDB: 1rbl A/ 154-462
PF00026	PEP2B_GADMO/13-323 PDB: 1am5 / 13-325
PF00036	OBL_OBELO/116-142 PDB: 1qv0 A/ 116-142
PF00044	G3P_METFE/2-140 PDB: 1cf2 P/ 2-140
PF00062	LYSC1_HORSE/1-127 PDB: 2eql / 1-127
PF00064	NRAM_INBBE/1-465 PDB: 1a4g B/ 76-465
PF00067	CPXB_BACME/7-449 PDB: 1bu7 A/ 7-449
PF00068	PA2GA_HUMAN/21-144 PDB: 1n29 A/ 2-124
PF00069	PHKG1_RABIT/20-288 PDB: 2phk A/ 20-288
PF00074	RNAS4_HUMAN/30-147 PDB: 2rnf B/ 2-119
PF00075	RNH_ECOLI/2-142 PDB: 1rdd / 2-142
PF00077	POL_HV1H2/492-586 PDB: 1a30 B/ 5-99
PF00080	SODC_YEAST/1-154 PDB: 2jcw / 1-153
PF00082	SUBT_BACLI/111-370 PDB: 1sca / 6-266
PF00085	PDIA1_HUMAN/25-132 PDB: 1mek / 8-115
PF00089	PLMN_HUMAN/581-803 PDB: 1ddj A/ 562-784
PF00102	PTN1_HUMAN/40-276 PDB: 1bzc A/ 40-276
PF00106	T4HR_MAGGR/30-197 PDB: 1ybv B/ 31-198
PF00109	FABB_ECOLI/2-246 PDB: 1dd8 D/ 2-246
PF00111	ADX_BOVIN/70-156 PDB: 1e6e D/ 12-98
PF00112	CATH_PIG/116-332 PDB: 8pch A/ 1-210
PF00115	CYOB_ECOLI/47-505 PDB: 1fft A/ 47-505
PF00117	TRPG_SULSO/5-195 PDB: 1qdl B/ 5-195
PF00118	THSB_THEAC/33-524 PDB: 1a6d B/ 33-521
PF00119	ATP6_ECOLI/39-266 PDB: 1c17 M/ 95-266

PF00121	TPIS_HUMAN/7-245 PDB: 1hti B/ 7-245
PF00127	PLAS_PROHO/37-131 PDB: 2b3i A/ 3-97
PF00135	PNBA_BACSU/2-485 PDB: 1qe3 A/ 2-485
PF00141	PER1A_ARMRU/48-299 PDB: 7atj A/ 18-269
PF00144	Q9KX40_9BURK/22-391 PDB: 1ci8 A/ 22-391
PF00145	MTH1_HAEPH/12-320 PDB: 1mht A/ 12-320
PF00149	USHA_ECOLI/34-256 PDB: 1ush / 34-256
PF00150	EXG_CANAL/94-233 PDB: 1cz1 A/ 56-195
PF00151	LIPP_HORSE/13-348 PDB: 1hpl B/ 1-335
PF00155	1A1C_MALDO/40-425 PDB: 1b8g A/ 40-425
PF00156	HPRT_HUMAN/33-164 PDB: 1bzy A/ 33-164
PF00161	ABRA_ABRPR/2-251 PDB: 1abr A/ 2-251
PF00162	PGKC_TRYBB/2-416 PDB: 13pk C/ 5-416
PF00171	BADH_GADCA/29-492 PDB: 1a4s C/ 29-492
PF00175	NCPR_RAT/530-642 PDB: 1amo A/ 531-643
PF00180	LEU3_THIFE/3-350 PDB: 1a05 B/ 3-350
PF00182	CHI2_HORVU/28-259 PDB: 1cns A/ 5-236
PF00186	DYR_ECOLI/1-158 PDB: 1ra2 / 1-158
PF00190	OXDC_BACSU/228-369 PDB: 1uw8 A/ 228-369
PF00194	CAH2_HUMAN/5-259 PDB: 1ca3 / 6-260
PF00202	DGDA_BURCE/28-367 PDB: 1d7r A/ 29-368
PF00205	MDLC_PSEPU/190-325 PDB: 1bfd / 190-325
PF00206	PUR8_THEMA/3-286 PDB: 1c3c A/ 3-286
PF00211	ADCY2_RAT/877-1077 PDB: 1ab8 A/ 877-1076
PF00215	PYRF_BACSU/4-230 PDB: 1dbt A/ 4-230
PF00221	HUTH_PSEPU/3-474 PDB: 1b8f A/ 3-474
PF00225	KINH_NEUCR/13-331 PDB: 1goj A/ 13-331
PF00232	LACG_LACLA/1-467 PDB: 1pbg A/ 1-467
PF00240	NEDD8_HUMAN/6-74 PDB: 2bkr B/ 6-74
PF00245	PPB_ECOLI/64-470 PDB: 1alk B/ 42-448
PF00246	CBPA1_BOVIN/128-406 PDB: 1cbx / 18-296

PF00248	ALDR_HUMAN/7-294 PDB: 2acu / 7-294
PF00254	FKB1A_HUMAN/11-105 PDB: 1d6o A/ 11-105
PF00255	GPX1_BOVIN/19-133 PDB: 1gp1 B/ 12-126
PF00264	PPO1_IPOBA/78-287 PDB: 1bt1 A/ 78-287
PF00266	P83788_PSEFL/30-392 PDB: 1qz9 A/ 30-392
PF00274	ALDOA_HUMAN/15-364 PDB: 1ald / 15-363
PF00275	MURA_ECOLI/6-406 PDB: 1uae / 6-406
PF00285	CISY_CHICK/44-422 PDB: 1al6 / 44-422
PF00288	ISPE_ECOL6/91-149 PDB: 1oj4 B/ 91-149
PF00290	TRPA_PYRFU/2-247 PDB: 1geq B/ 2-247
PF00291	CYSK_SALTY/8-301 PDB: 1oas A/ 8-301
PF00293	O33199_MYCTU/42-176 PDB: 1mqw A/ 42-176
PF00294	RBSK_ECOLI/4-298 PDB: 1rk2 C/ 4-298
PF00295	PGLR2_ASPNG/45-362 PDB: 1czf A/ 45-362
PF00296	LUXB_VIBHA/1-323 PDB: 1luc B/ 1-320
PF00300	PMG1_YEAST/3-189 PDB: 1qhf A/ 3-189
PF00302	CAT3_ECOLX/1-205 PDB: 3cla / 6-211
PF00303	TYSA_BACSU/3-279 PDB: 1b02 A/ 3-279
PF00310	ASNB_ECOLI/2-162 PDB: 1ct9 B/ 2-162
PF00311	CAPP_ECOLI/1-883 PDB: 1qb4 A/ 1-883
PF00316	F16P1_PIG/12-335 PDB: 1eyi A/ 12-335
PF00326	PPCE_PIG/482-707 PDB: 1qfm A/ 482-707
PF00328	PPAP_RAT/33-331 PDB: 1rpt / 2-300
PF00331	GUX_CELFI/44-353 PDB: 2his / 3-312
PF00332	GUB2_HORVU/7-312 PDB: 1aq0 A/ 1-306
PF00334	NDKC_DICDI/9-143 PDB: 1nsp / 9-143
PF00342	G6PI_RABIT/54-546 PDB: 1dqr B/ 54-546
PF00343	PYGM_RABIT/112-830 PDB: 1gpa D/ 112-830
PF00348	FPPS_CHICK/58-330 PDB: 1fps / 58-330
PF00349	HXKB_YEAST/21-223 PDB: 1ig8 A/ 22-224
PF00355	NDOB_PSEPU/38-139 PDB: 1ndo A/ 38-139

PF00358 PTG3C_BACSU/547-679 PDB: 1gpr / 10-142

PF00365 K6PF1_ECOLI/3-278 PDB: 1pfk A/ 2-277

PF00378 ECH1_RAT/67-247 PDB: 1dci A/ 67-247

PF00383 CDD_ECOLI/47-149 PDB: 1ctt / 47-149

PF00388 PLC_LISMO/61-200 PDB: 2plc / 39-178

PF00389 SERA_ECOLI/13-326 PDB: 1psd A/ 14-327

PF00390 MAOM_HUMAN/89-278 PDB: 1do8 B/ 89-278

PF00398 ERM_BACSU/1-244 PDB: 1qam A/ 1-244

PF00413 MMP2_HUMAN/118-446 PDB: 1qib A/ 91-235

PF00432 SQHC_ALIAC/514-567 PDB: 2sqc A/ 515-568

PF00445 RNRH_RHINI/32-227 PDB: 1bol A/ 16-211

PF00450 CBP2_WHEAT/15-433 PDB: 1bcr B/ 264-422

PF00457 XYNA_BACCI/27-212 PDB: 1bvv / 1-184

PF00459 HAL2_YEAST/41-353 PDB: 1qgx A/ 41-353

PF00462 GLRX1_ECOLI/3-69 PDB: 1qfn A/ 3-69

PF00463 ACEA_MYCTU/9-427 PDB: 1f8m A/ 9-427

PF00464 GLYA_ECOLI/8-386 PDB: 1dfo D/ 8-386

PF00479 G6PD_LEUME/10-188 PDB: 1dpg B/ 10-188

PF00484 CAN_ECOLI/30-192 PDB: 1i6p A/ 30-192

PF00490 HEM2_YEAST/13-338 PDB: 1h7o A/ 13-338

PF00491 ARGI_BACCD/2-295 PDB: 1cev D/ 2-295

PF00501 LUCI_PHOPY/52-469 PDB: 1lci / 52-469

PF00503 GNAI1_RAT/6-353 PDB: 1bh2 / 32-346

PF00521 GYRA_MYCXE/1-313 PDB: 1am2 / 2-198

PF00540 POL_HV1H2/2-132 PDB: 1hiv B/ 1-76

PF00544 PELC_ERWCH/71-279 PDB: 2pec / 49-257

PF00545 RNAS_ASPGI/83-175 PDB: 1de3 A/ 56-148

PF00548 POLG_HRV2/1508-1673 PDB: 1cqq A/ 2-167

PF00550 ACP_BACSU/6-73 PDB: 1f80 F/ 5-72

PF00551 PUR3_ECOLI/1-181 PDB: 1c2t A/ 1-181

PF00557 AMPP_ECOLI/183-422 PDB: 1a16 / 183-422

PF00561	LINB_PSEPA/56-288 PDB: 1cv2 A/ 57-289
PF00565	NUC_STAAU/90-224 PDB: 1a2t / 8-141
PF00574	CLPP_ECOLI/25-206 PDB: 1tyf E/ 11-192
PF00579	SYW_BACST/1-281 PDB: 1d2r B/ 1-281
PF00581	THTR_AZOVI/12-123 PDB: 1e0c A/ 12-123
PF00583	SNAT_SHEEP/84-174 PDB: 1b6b A/ 84-174
PF00587	SYS_THET2/168-340 PDB: 1ses B/ 168-340
PF00590	CYSG_SALTY/217-427 PDB: 1pjq B/ 217-427
PF00596	FUCA_ECOLI/8-182 PDB: 1fua / 8-182
PF00607	Q72497_9HIV1/149-363 PDB: 1m9c D/ 17-146
PF00632	UBE3A_HUMAN/576-875 PDB: 1c4z B/ 553-852
PF00633	END3_ECOLI/99-128 PDB: 2abk / 99-128
PF00639	PIN1_HUMAN/59-163 PDB: 1nmw A/ 59-163
PF00648	CAN2_HUMAN/45-344 PDB: 1kfu L/ 46-345
PF00656	CASP9_HUMAN/161-413 PDB: 1nw9 B/ 161-413
PF00657	PA1B3_BOVIN/41-208 PDB: 1bwp / 41-208
PF00677	RISA_SCHPO/3-87 PDB: 1kzl A/ 3-87
PF00682	DMPG_PSEUF/17-273 PDB: 1nvm G/ 17-273
PF00685	ST1E1_HUMAN/37-287 PDB: 1hy3 A/ 37-287
PF00692	DUT_ECOLI/15-149 PDB: 1dup A/ 16-136
PF00694	ACON_BOVIN/582-712 PDB: 1fgh / 555-685
PF00696	ARGB_ECOLI/3-236 PDB: 1oh9 A/ 3-236
PF00697	TRPF_THEMA/4-201 PDB: 1nsj / 4-201
PF00698	FABD_ECOLI/61-227 PDB: 1mla / 62-228
PF00701	NANA_ECOLI/4-293 PDB: 1fdy A/ 5-294
PF00702	HAD_PSEUY/4-197 PDB: 1qh9 A/ 4-197
PF00704	CHIA_SERMA/158-544 PDB: 1ctn / 158-544
PF00708	ACYP1_BOVIN/3-100 PDB: 2acy / 1-98
PF00709	PURA_ECOLI/4-425 PDB: 1gim / 4-425
PF00710	ASPQ_PSES7/11-331 PDB: 1djo B/ 3011-3331
PF00716	VP40_HCMVA/31-415 PDB: 1nkk C/ 1031-1256

PF00717	LEP_ECOLI/86-170 PDB: 1t7d A/ 85-169
PF00719	IPYR_YEAST/45-229 PDB: 1wgi B/ 45-229
PF00722	GUB_PAEMA/49-233 PDB: 2ayh / 26-210
PF00723	AMYG_ASPKA/36-450 PDB: 1agm A/ 12-427
PF00724	OYE1_SACPS/16-368 PDB: 1bwl A/ 16-368
PF00725	HCDH_HUMAN/216-313 PDB: 2hdh B/ 204-301
PF00730	3MG2_ECOLI/118-262 PDB: 1diz A/ 118-262
PF00749	SYE_THET8/1-312 PDB: 1j09 A/ 1-312
PF00753	GLO2_HUMAN/11-173 PDB: 1qh5 A/ 11-173
PF00755	CLAT_RAT/22-612 PDB: 1q6x B/ 23-613
PF00759	GUN4_THEFU/51-484 PDB: 1js4 A/ 5-438
PF00762	HEMH_HUMAN/68-389 PDB: 1hrk B/ 68-389
PF00770	ADEN_ADE02/20-202 PDB: 1nln A/ 20-202
PF00793	KDSA_ECOLI/10-276 PDB: 1q3n A/ 10-276
PF00795	DCAS_AGRSK/6-197 PDB: 1uf7 A/ 5-196
PF00797	NHOA_SALTY/21-257 PDB: 1e2t D/ 21-257
PF00809	DHPS_ECOLI/20-228 PDB: 1aj0 / 20-228
PF00815	HISX_ECOLI/17-428 PDB: 1kae B/ 18-429
PF00821	PPCKC_HUMAN/29-622 PDB: 1nhx A/ 29-622
PF00834	RPE_SOLTU/58-260 PDB: 1rpx B/ 12-214
PF00840	GUX1_TRIRE/19-449 PDB: 1cel B/ 2-432
PF00857	CSH_ARTSP/43-229 PDB: 1nba A/ 43-229
PF00871	ACKA_METTE/2-390 PDB: 1g99 B/ 2-390
PF00881	NFNB_ECOLI/7-193 PDB: 1ylu A/ 7-193
PF00882	PHLC_BACCE/1-282 PDB: 1ah7 / 1-244
PF00884	ARSA_HUMAN/19-440 PDB: 1auk / 19-440
PF00885	RISB_BACSU/10-153 PDB: 1rvv A/ 10-153
PF00890	FRDA_SHEON/152-575 PDB: 1d4c A/ 127-550
PF00891	COMT1_MEDSA/95-340 PDB: 1kyw C/ 95-340
PF00903	LGUL_HUMAN/31-174 PDB: 1fro C/ 31-174
PF00905	BLO1_ECOLX/42-276 PDB: 1m6k B/ 36-274

PF00908	RFBC_SALTY/4-179 PDB: 1dzr B/ 4-179
PF00923	TALB_ECOLI/13-313 PDB: 1onr A/ 14-314
PF00926	RIBB_METJA/4-222 PDB: 1snn A/ 4-222
PF00929	THEX1_HUMAN/130-306 PDB: 1w0h A/ 131-307
PF00962	ADA_MOUSE/8-346 PDB: 1a4l C/ 1009-1347
PF00975	ERYA3_SACER/2958-3167 PDB: 1kez B/ 70-279
PF00977	HIS6_THEMA/5-233 PDB: 2a0n A/ 5-233
PF00982	OTSA_ECOLI/2-453 PDB: 1uqt A/ 2-453
PF01028	TOP1_VACCV/63-305 PDB: 1a41 / 81-305
PF01039	Q9X4K7_STRCO/35-528 PDB: 1xny A/ 35-528
PF01041	O52552_AMYMD/14-381 PDB: 1b9h A/ 14-381
PF01048	MTAP_HUMAN/11-256 PDB: 1cg6 A/ 11-256
PF01053	Q9ZPL5_TOBAC/58-443 PDB: 1qgn D/ 58-443
PF01058	PHSS_DESBA/50-210 PDB: 1cc1 S/ 18-178
PF01063	DAAA_BACYM/43-273 PDB: 1daa A/ 43-273
PF01068	DNLI_BPT7/8-240 PDB: 1a0i / 8-240
PF01070	CYB2_YEAST/212-560 PDB: 1fcb A/ 132-480
PF01077	CYSI_ECOLI/172-331 PDB: 1aop / 173-332
PF01081	ALKH_ECOLI/8-203 PDB: 1fq0 C/ 8-203
PF01083	AXE2_PENPU/27-234 PDB: 1bs9 / 1-207
PF01088	UBL1_YEAST/7-218 PDB: 1cmx C/ 407-618
PF01095	PME_DAUCA/8-306 PDB: 1gq8 A/ 8-306
PF01112	ASPG_HUMAN/8-335 PDB: 1apy D/ 183-312
PF01116	ALF_ECOLI/14-359 PDB: 1b57 B/ 14-358
PF01126	HMUO_CORDI/6-210 PDB: 1wnw C/ 6-210
PF01129	NAR2B_RAT/27-239 PDB: 1og1 A/ 7-219
PF01135	PIMT_THEMA/1-213 PDB: 1dl5 B/ 1-213
PF01149	FPG_ECOLI/2-115 PDB: 1k82 D/ 2-115
PF01156	IUNH_CRIFA/2-312 PDB: 1mas A/ 3-313
PF01168	ALR_BACST/12-229 PDB: 1bd0 B/ 12-229
PF01177	MURI_AQUPY/2-213 PDB: 1b73 A/ 2-213

PF01179 AMO_ECOLI/331-757 PDB: 1oac B/ 301-727

PF01180 PYRD_HUMAN/77-377 PDB: 1d3g A/ 78-378

PF01182 NAGB_ECOLI/15-250 PDB: 1cd5 A/ 15-250

PF01188 CATB_PSEPU/149-247 PDB: 1muc B/ 148-246

PF01195 PTH_ECOLI/5-189 PDB: 2pth / 4-188

PF01208 DCUP_HUMAN/14-360 PDB: 1uro A/ 14-360

PF01212 TNAA_PROVU/47-433 PDB: 1ax4 A/ 47-433

PF01220 AROQ_ACTPL/2-145 PDB: 1uqr E/ 2-145

PF01223 NUCA_SERMA/45-246 PDB: 1smn A/ 24-225

PF01227 GCH1_ECOLI/98-202 PDB: 1gtp L/ 98-202

PF01230 FHIT_HUMAN/8-105 PDB: 5fit / 9-106

PF01238 MANA_CANAL/6-398 PDB: 1pmi / 7-399

PF01242 PTPS_RAT/2-144 PDB: 1b66 B/ 7-144

PF01244 DPEP1_HUMAN/25-352 PDB: 1itq A/ 9-336

PF01255 UPPS_MICLU/27-248 PDB: 1f75 A/ 27-248

PF01261 XYLA_STRRU/40-265 PDB: 2xis / 41-266

PF01263 GALM_HUMAN/20-338 PDB: 1snz A/ 20-338

PF01266 OXDA_RHOTO/6-351 PDB: 1c0k A/ 1006-1351

PF01268 FTHS_MOOTH/4-559 PDB: 1eg7 A/ 1004-1559

PF01274 MASZ_ECOLI/17-697 PDB: 1d8c A/ 18-698

PF01276 DCOR_LACS3/109-569 PDB: 1ord B/ 109-569

PF01278 OMPT_ECOLI/21-317 PDB: 1i78 A/ 1-297

PF01288 HPPK_ECOLI/5-133 PDB: 1hka / 5-133

PF01293 PPCK_ECOLI/19-493 PDB: 1aq2 / 19-493

PF01327 DEF_ECOLI/3-153 PDB: 1bs4 B/ 503-653

PF01328 PRXC_CALFU/1-317 PDB: 2cpo / 1-296

PF01329 PHS_RAT/2-101 PDB: 1dco D/ 6-102

PF01339 CHEB_SALTY/158-340 PDB: 1chd / 158-340

PF01341 GUX2_TRIRE/124-437 PDB: 1qk2 A/ 100-413

PF01344 GAOA_GIBZE/311-357 PDB: 1gog / 270-316

PF01361 4OT1_PSEPU/2-61 PDB: 1bjp E/ 2-61

PF01364	CPG2_PORGI/230-578 PDB: 1cvr A/ 1-349
PF01370	SQD1_ARATH/87-377 PDB: 1qrr A/ 4-294
PF01373	AMYB_SOYBN/16-438 PDB: 1bya / 16-438
PF01374	CHIS_STRSN/55-266 PDB: 1chk A/ 15-226
PF01376	CHTB_VIBCH/22-123 PDB: 1xtc H/ 1-102
PF01400	ASTA_ASTFL/57-218 PDB: 1ast / 8-169
PF01401	ACET_HUMAN/60-654 PDB: 1o8a A/ 37-623
PF01408	GFO_ZYMMO/84-208 PDB: 1ofg B/ 32-156
PF01425	FAAH1_RAT/95-562 PDB: 1mt5 P/ 95-562
PF01427	VANX_ENTFC/1-202 PDB: 1r44 E/ 1-202
PF01432	NEUL_RAT/251-701 PDB: 1i1i P/ 228-678
PF01433	LKHA4_HUMAN/21-387 PDB: 1h19 A/ 21-387
PF01447	THER_BACTH/236-383 PDB: 1tlp E/ 4-151
PF01451	PPAL_YEAST/8-159 PDB: 1d1q A/ 7-158
PF01467	COAD_ECOLI/6-135 PDB: 1b6t A/ 6-135
PF01470	PCP_BACAM/3-204 PDB: 1aug B/ 213-414
PF01487	AROD_SALTI/18-245 PDB: 1qfe A/ 18-245
PF01494	PH2M_TRICU/8-416 PDB: 1foh A/ 8-416
PF01501	P96945_NEIME/2-256 PDB: 1ga8 A/ 2-256
PF01504	PI42B_HUMAN/119-415 PDB: 1bo1 A/ 119-415
PF01510	NAAA_BPT7/2-146 PDB: 1lba / 6-146
PF01532	MNS1_YEAST/45-532 PDB: 1dl2 A/ 45-532
PF01546	CBPG_PSES6/108-410 PDB: 1cg2 B/ 108-410
PF01547	THI1_PANTH/46-330 PDB: 2thi A/ 16-300
PF01553	PLSB_CUCMO/146-305 PDB: 1k30 A/ 118-277
PF01555	MTP2_PROVU/47-305 PDB: 1boo A/ 47-305
PF01565	DH4C_PSEPU/58-198 PDB: 1dii B/ 59-199
PF01569	PRXV_ASCNO/318-514 PDB: 1qi9 B/ 318-514
PF01575	DHB4_HUMAN/480-601 PDB: 1s9c E/ 163-284
PF01593	PAO_MAIZE/42-483 PDB: 1b5q A/ 14-455
PF01596	COMT_RAT/56-229 PDB: 1vid / 13-186

PF01625	MSRA_ECOLI/44-203 PDB: 1ff3 A/ 44-203
PF01630	HUGA_APIME/36-368 PDB: 1fcq A/ 4-336
PF01636	KKA3_ENTFA/18-257 PDB: 1l8t A/ 18-257
PF01640	SPEB_STRPY/12-398 PDB: 1dki A/ -10-253
PF01641	MSRAB_NEIGO/383-506 PDB: 1l1d A/ 383-506
PF01642	MUTA_PROFR/211-485 PDB: 1req B/ 212-486
PF01648	ACPS_BACSU/5-73 PDB: 1f7l A/ 5-73
PF01653	DNLJ_THEFI/4-319 PDB: 1dgs B/ 2004-2316
PF01656	BIOD_ECOLI/5-218 PDB: 1dae / 5-218
PF01670	Q54331_STRLI/111-261 PDB: 2nlr A/ 71-221
PF01674	LIP_BACSU/34-197 PDB: 1r4z B/ 3-166
PF01678	DAPF_HAEIN/151-267 PDB: 1bwz A/ 151-267
PF01712	DGUOK_HUMAN/110-267 PDB: 1jag E/ 110-267
PF01729	NADC_MYCTU/117-284 PDB: 1qpr A/ 117-284
PF01739	CHER_SALTY/92-283 PDB: 1af7 / 92-283
PF01746	TRMD_HAEIN/22-221 PDB: 1uam A/ 22-221
PF01747	MET3_YEAST/72-388 PDB: 1j70 A/ 72-388
PF01751	TOP3_ECOLI/1-135 PDB: 1d6m A/ 1-135
PF01761	ARO1_EMENI/26-359 PDB: 1dqs B/ 26-359
PF01791	DEOC_ECOLI/10-246 PDB: 1p1x B/ 1010-1246
PF01804	PAC_ECOLX/31-838 PDB: 1pnl A/ 5-195
PF01812	Y348_MYCPN/3-163 PDB: 1u3f B/ 3-163
PF01817	CHMU_YEAST/16-85 PDB: 3csm A/ 16-85
PF01820	DDLB_ECOLI/3-95 PDB: 2dln / 4-96
PF01828	PRTB_SCYLI/57-260 PDB: 1s2k A/ 3-206
PF01844	CEA9_ECOLX/536-582 PDB: 1fr2 B/ 88-134
PF01853	ESA1_YEAST/220-406 PDB: 1mj9 A/ 220-406
PF01862	PDAD_METJA/13-165 PDB: 1mt1 F/ 54-165
PF01880	SOR_PYRFU/8-120 PDB: 1do6 A/ 8-120
PF01915	Q9XEI3_HORVD/414-626 PDB: 1ex1 A/ 389-601
PF01916	DHYS_HUMAN/42-356 PDB: 1roz B/ 42-356

PF01948	PYRI_ECOLI/4-99 PDB: 1at1 D/ 8-100
PF01979	URE1_KLEAE/125-436 PDB: 1kra C/ 125-436
PF02089	PPT2_HUMAN/32-302 PDB: 1pja A/ 32-302
PF02102	NPII_ASPOR/1-352 PDB: 1eb6 A/ 1-177
PF02126	OPD_BREDI/44-359 PDB: 1ez2 B/ 44-359
PF02136	SDIS_PSEPU/8-128 PDB: 1vzz A/ 8-128
PF02142	MGSA_ECOLI/25-118 PDB: 1b93 C/ 25-118
PF02146	HST2_YEAST/32-230 PDB: 1szd A/ 32-230
PF02152	FOLB_STAAU/5-120 PDB: 2dhn / 5-120
PF02222	PURT_ECOLI/113-295 PDB: 1ez1 A/ 114-296
PF02230	EST2_PSEFL/1-217 PDB: 1auo B/ 1-217
PF02233	NNTM_HUMAN/618-1080 PDB: 1djl A/ 837-1037
PF02237	BIRA_ECOLI/271-317 PDB: 1bib / 271-317
PF02253	PA1_ECOLI/22-286 PDB: 1qd6 C/ 30-266
PF02255	PTLA_LACLA/7-102 PDB: 1e2a B/ 7-102
PF02261	PAND_ECOLI/1-116 PDB: 1aw8 E/ 26-115
PF02265	NUP1_PENCI/1-265 PDB: 1ak0 / 1-264
PF02267	NADA_APLCA/28-272 PDB: 1r16 A/ 4-248
PF02273	LUXD_VIBHA/6-299 PDB: 1tht A/ 6-299
PF02275	PAC_BACSH/4-320 PDB: 3pva H/ 1-317
PF02277	COBT_SALTY/7-346 PDB: 1d0s A/ 7-346
PF02288	Q59471_KLEOX/48-222 PDB: 1dio B/ 48-222
PF02310	MAMA_CLOTT/4-130 PDB: 1be1 / 4-130
PF02329	DCHS_LACS3/5-310 PDB: 1pya C/ 5-81
PF02332	MEMB_METTR/74-311 PDB: 1mhy B/ 75-312
PF02350	WECB_ECOLI/21-370 PDB: 1f6d A/ 21-370
PF02374	ARSA1_ECOLX/327-583 PDB: 1f48 A/ 327-583
PF02388	Q9EY50_LACVI/8-335 PDB: 1p4n A/ 7-334
PF02423	Q88H32_PSEPK/7-315 PDB: 1x7d A/ 7-315
PF02431	CFI1_MEDSA/15-212 PDB: 1eyp B/ 15-212
PF02435	SACB_BACSU/37-470 PDB: 1oyg A/ 37-470

PF02441	HAL3A_ARATH/20-140 PDB: 1mvn A/ 20-140
PF02446	MALQ_THETH/11-497 PDB: 1cwy A/ 11-497
PF02502	RPIB_ECOLI/3-142 PDB: 1nn4 A/ 3-142
PF02515	FCTA_OXAFO/70-292 PDB: 1t4c A/ 71-293
PF02518	SP2AB_BACST/35-139 PDB: 1l0o A/ 35-136
PF02525	NQO1_HUMAN/4-217 PDB: 1d4a A/ 3-216
PF02560	CYNS_ECOLI/83-156 PDB: 1dw9 G/ 83-156
PF02570	COBH_PSEDE/10-210 PDB: 1f2v A/ 10-210
PF02572	BTUR_SALTY/22-196 PDB: 1g64 A/ 22-196
PF02580	DTD_HAEIN/2-144 PDB: 1j7g A/ 2-144
PF02581	THIE_BACSU/15-198 PDB: 2tps A/ 28-211
PF02615	DLGD_ECOLI/1-332 PDB: 1s20 E/ 1-332
PF02633	P83772_PSEPU/5-258 PDB: 1j2u B/ 5-258
PF02668	TAUD_ECOLI/6-280 PDB: 1os7 D/ 7-281
PF02685	GLK_ECOLI/6-316 PDB: 1q18 B/ 6-316
PF02709	B4GT1_BOVIN/134-402 PDB: 1fr8 B/ 134-402
PF02729	OTC1_ECOLI/7-148 PDB: 1akm A/ 7-148
PF02779	ODPB_HUMAN/31-208 PDB: 1ni4 D/ 2-178
PF02784	DCOR_MOUSE/44-282 PDB: 7odc A/ 44-282
PF02797	CHS2_MEDSA/238-388 PDB: 1cgk A/ 238-388
PF02798	PTGD2_RAT/4-73 PDB: 1pd2 2/ 5-74
PF02803	THIL_ZOORA/269-391 PDB: 1qfl B/ 270-392
PF02826	DHGY_HYPME/111-290 PDB: 1gdh A/ 111-290
PF02866	MDH_ECOLI/147-310 PDB: 1emd / 147-310
PF02878	ALGC_PSEAE/11-145 PDB: 1p5d X/ 12-146
PF02896	PPDK_CLOSY/515-871 PDB: 1kc7 A/ 516-872
PF02900	PCYB_PSEPA/6-274 PDB: 1bou B/ 6-274
PF02901	PFLB_ECOLI/11-614 PDB: 2pfl A/ 11-614
PF02907	POLG_HCVH/1057-1204 PDB: 1rgq B/ 35-182
PF02940	CET1_YEAST/278-497 PDB: 1d8h A/ 278-497
PF02945	END7_BPT4/1-97 PDB: 1e7l A/ 1-97

PF02962	HPCD_ECOLX/2-125 PDB: 1otg B/ 3-126
PF02979	NHAA_RHOER/5-202 PDB: 2ahj A/ 5-202
PF02982	SCYD_MAGGR/10-169 PDB: 1std / 10-169
PF03013	END5_BPT4/1-138 PDB: 1vas A/ 2-138
PF03051	BLH1_YEAST/33-479 PDB: 1gcb / 33-453
PF03071	MGAT1_RABIT/12-446 PDB: 1foa A/ 106-446
PF03098	PERM_HUMAN/173-718 PDB: 1mhl D/ 113-552
PF03150	P83787_MARHY/27-181 PDB: 1nml A/ 27-181
PF03167	UNG_ECOLI/49-211 PDB: 1eug A/ 50-212
PF03171	IPNS_EMENI/180-288 PDB: 1qje A/ 180-288
PF03241	HDVD_CLOAM/109-489 PDB: 1u8v A/ 109-489
PF03328	GARL_ECOLI/20-246 PDB: 1dxe B/ 20-246
PF03352	3MG1_ECOLI/6-184 PDB: 1p7m A/ 6-184
PF03360	B3GA3_HUMAN/96-313 PDB: 1kws A/ 96-313
PF03372	APEX1_HUMAN/62-316 PDB: 1bix / 63-317
PF03414	GGTA1_BOVIN/39-366 PDB: 1vzx B/ 1080-1366
PF03435	LYS9_MAGGR/6-445 PDB: 1e5q H/ 6-445
PF03441	PHR_ECOLI/203-469 PDB: 1dnp A/ 202-468
PF03446	6PGD_SHEEP/3-176 PDB: 2pgd / 3-176
PF03572	TRI_THEAC/878-1033 PDB: 1k32 B/ 878-1033
PF03575	PEPE_SALTY/49-202 PDB: 1fy2 A/ 49-202
PF03576	Q59632_OCHAN/26-366 PDB: 1b65 B/ 26-366
PF03590	ASNA_ECOLI/3-246 PDB: 12as A/ 4-246
PF03712	AMD_RAT/199-352 PDB: 1opm A/ 199-352
PF03718	DEXT_PENMI/26-608 PDB: 1ogo X/ 2-574
PF03721	UDG_STRPY/1-186 PDB: 1dli A/ 1-186
PF03852	VSR_ECOLI/2-76 PDB: 1cw0 A/ 3-77
PF03900	HEM3_ECOLI/225-298 PDB: 2ypn A/ 225-298
PF03917	GSHB_HUMAN/5-474 PDB: 2hgs A/ 5-474
PF03936	ARIS_PENRO/43-293 PDB: 1di1 A/ 43-292
PF04055	BIOB_ECOLI/47-209 PDB: 1r30 B/ 47-209

PF04127	COABC_ECOLI/186-386 PDB: 1u7u A/ 187-387
PF04209	HGD_HUMAN/3-434 PDB: 1ey2 A/ 3-434
PF04389	AMPX_VIBPR/196-377 PDB: 1amp / 90-271
PF04828	GFA_PARDE/50-132 PDB: 1xa8 D/ 53-135
PF04928	PAP_YEAST/4-352 PDB: 1fa0 A/ 4-352
PF05014	NTD_LACLE/6-134 PDB: 1f8x B/ 207-335
PF05067	MCAT_LACPL/1-246 PDB: 1o9i F/ 1-246
PF05173	DAPB_ECOLI/132-268 PDB: 1arz A/ 132-268
PF05175	Q9WYV8_THEMA/125-199 PDB: 1vq1 A/ 125-199
PF05191	KAD_BACST/127-162 PDB: 1zio / 127-162
PF05199	GOX_ASPNG/452-593 PDB: 1gal / 430-571
PF05201	HEM1_METKA/7-142 PDB: 1gpj A/ 7-142
PF05221	SAHH_RAT/5-431 PDB: 1b3r A/ 5-431
PF05222	O52942_PHOLP/4-136 PDB: 1pjb A/ 4-136
PF05351	PDE6D_HUMAN/7-150 PDB: 1ksj B/ 7-150
PF05409	R1AB_CVHSA/3269-3565 PDB: 2bx4 A/ 29-306
PF05426	Q9KWU1_9SPHN/30-387 PDB: 1qaz A/ 6-339
PF05448	P94388_BACSU/1-316 PDB: 1odt H/ 1-316
PF06002	Q9LAK3_CAMJE/1-291 PDB: 1ro7 C/ 1-259
PF06134	RHAA_ECOLI/3-418 PDB: 1de6 C/ 11-426
PF06433	DHMH_PARDE/74-415 PDB: 2bbk H/ 30-371
PF06441	O31243_9RHIZ/9-49 PDB: 1ehy B/ 9-49
PF06442	DYR21_ECOLX/1-78 PDB: 1vie / 19-78
PF06941	NT5M_HUMAN/34-227 PDB: 1q91 A/ 34-227
PF07714	INSR_HUMAN/1023-1290 PDB: 1ir3 A/ 996-1263
PF07722	GGH_HUMAN/34-246 PDB: 1l9x B/ 10-222
PF07732	NIR_ACHCY/82-199 PDB: 1nid / 44-161
PF07736	CHMU_BACSU/3-120 PDB: 1dbf C/ 3-120
PF07745	GANA_ASPAC/20-347 PDB: 1fob A/ 4-331
PF07823	CPD_ARATH/1-179 PDB: 1jh6 A/ 1-179
PF07832	T2CX_CITFR/1-285 PDB: 1cfr / 1-283

PF07858	LIMA_RHOER/22-145 PDB: 1nww B/ 23-146
PF07882	FUCI_ECOLI/175-355 PDB: 1fui A/ 175-355
PF07969	CODA_ECOLI/53-379 PDB: 1ra0 A/ 53-379
PF07977	FABA_ECOLI/29-159 PDB: 1mka B/ 29-159
PF07992	GSHR_ECOLI/6-309 PDB: 1get A/ 6-309
PF08240	ETR1_CANTR/55-146 PDB: 1guf A/ 55-146
PF08242	GNMT_RAT/62-171 PDB: 1d2h C/ 62-171
PF08245	MURD_ECOLI/108-280 PDB: 1uag / 108-280
PF08543	THID_SALTY/13-260 PDB: 1jxh A/ 13-260
PF09000	CEA3_ECOLX/464-551 PDB: 1jch C/ 464-551
PF09017	TGAS_STRMB/1-407 PDB: 1iu4 D/ 1-331
PF09056	Q9Z4W2_STRCO/42-151 PDB: 1it4 A/ 13-122
PF09113	PNGF_ELIMR/181-352 PDB: 1pgs / 141-312
PF09198	GSTB_BPT4/1-38 PDB: 1c3j A/ 1-38
PF09225	T2P2_PROVU/3-157 PDB: 1pvi A/ 3-157
PF09254	T2F1_FLAOK/391-583 PDB: 2fok B/ 387-579
PF09334	SYM_THET8/5-361 PDB: 1a8h / 5-361
PF09492	Q9X592_AZOIR/118-422 PDB: 1r76 A/ 118-422
PF09588	EXO_LAMBD/23-168 PDB: 1avq B/ 23-168
